# Undergraduate medical education in emergency medical care: A nationwide survey at German medical schools

**DOI:** 10.1186/1471-227X-9-7

**Published:** 2009-05-12

**Authors:** Stefan K Beckers, Arnd Timmermann, Michael P Müller, Matthias Angstwurm, Felix Walcher

**Affiliations:** 1 Section Emergency Medical Care, Department of Anaesthesiology, Medical Faculty RWTH Aachen University, Aachen, Germany; 2 AIXTRA – Aix-la-Chapelle Centre for Interdisciplinary Training in Medical Education, Aachen, Germany; 3 Department of Anaesthesiology, Emergency and Intensive Care Medicine, University Hospital Georg-August University, Göttingen, Germany; 4 Department of Anaesthesiology and Intensive Care Medicine, University of Technology, Dresden, Germany; 5 Department of Internal medicine, Medical faculty, Ludwig-Maximilians-University, Munich, Germany; 6 Department of Traumatology, University Hospital Frankfurt/Main, Germany; 7 Committee for Emergency Medical Care and Simulation, German Association for Medical Education, Germany

## Abstract

**Background:**

Since June 2002, revised regulations in Germany have required "Emergency Medical Care" as an interdisciplinary subject, and state that emergency treatment should be of increasing importance within the curriculum. A survey of the current status of undergraduate medical education in emergency medical care establishes the basis for further committee work.

**Methods:**

Using a standardized questionnaire, all medical faculties in Germany were asked to answer questions concerning the structure of their curriculum, representation of disciplines, instructors' qualifications, teaching and assessment methods, as well as evaluation procedures.

**Results:**

Data from 35 of the 38 medical schools in Germany were analysed. In 32 of 35 medical faculties, the local Department of Anaesthesiology is responsible for the teaching of emergency medical care; in two faculties, emergency medicine is taught mainly by the Department of Surgery and in another by Internal Medicine. Lectures, seminars and practical training units are scheduled in varying composition at 97% of the locations. Simulation technology is integrated at 60% (n = 21); problem-based learning at 29% (n = 10), e-learning at 3% (n = 1), and internship in ambulance service is mandatory at 11% (n = 4). In terms of assessment methods, multiple-choice exams (15 to 70 questions) are favoured (89%, n = 31), partially supplemented by open questions (31%, n = 11). Some faculties also perform single practical tests (43%, n = 15), objective structured clinical examination (OSCE; 29%, n = 10) or oral examinations (17%, n = 6).

**Conclusion:**

Emergency Medical Care in undergraduate medical education in Germany has a practical orientation, but is very inconsistently structured. The innovative options of simulation technology or state-of-the-art assessment methods are not consistently utilized. Therefore, an exchange of experiences and concepts between faculties and disciplines should be promoted to guarantee a standard level of education in emergency medical care.

## Background

### Regulations for undergraduate medical education in Germany

In June 2002, Germany revised nationwide regulations, requiring new subjects such as anaesthesiology or public health as compulsory subjects, or interdisciplinary courses in health economics, ethics or epidemiology within the different local curricula [1]. "Emergency Medical Care" was introduced as an interdisciplinary subject, because issues in emergency treatment are of increasing importance within the curriculum.

In general, these areas are "interdisciplinary" with an integrating character to various disciplines and are intended to prepare the professional for the practical requirements of working life as physician. With respect to "Emergency Medical Care," existing courses in two different parts of the curriculum were centralized and combined under one central theme. With respect to a detailed implementation of the new regulations, it was postulated that medical education in these areas has to be focused on patient care, related to practice and should integrate small group training sessions where possible [1].

Another innovation to be implemented nationwide was the request to assess and grade every subject within the curriculum, and to include these results in the final certificate. However, the duration and the modality of the examinations are not defined in detail. These requirements demanded the best effort from many faculties and disciplines, especially where methods and structures of routine assessments were not established.

Additionally, an appropriate standard for quality management in undergraduate education was set for the first time: all courses have to be evaluated regularly, and these results have to be published.

Unfortunately, the 2002 regulations did not specify the impact of the sustainability of the evaluation data.

As a result, all medical schools had to re-arrange courses and curricular structure, because of the general consequences of the new regulations, including a shift in the defined workload of the participating disciplines, implementation of new assessment requirements etc. [1].

The process itself was not defined nor regulated, such that the intensity and professionalism of the curriculum development depended substantially on local engagement, resources, and on the influence, power, and interests in teaching of the representatives of various disciplines within the local committees.

### Committee for "Emergency Medical Care and Simulation"

In 1998, Burdick et al. stated correctly that society has a right to expect that every physician is able to manage acute problems of patients and that a basic knowledge of emergency medical care has to exist [2]. Following this postulation, and based on the developments initiated through legal changes, the "Committee for Emergency Medical Care and simulation" was founded within the "German Association for Medical Education" (GMA) by medical professionals engaged in medical education and simulation [2,3]; their professional backgrounds – internal medicine, traumatology and anaesthesiology – emphasize the interdisciplinary approach of the committee.

The objective of this committee is to establish an interdisciplinary, nationwide forum for discussion, exchange of ideas and concepts of continuous improvement in education of emergency medical care as well as the implementation of simulation technology in this field. The committee is explicitly interdisciplinary and accessible for all interested professionals involved in education with respect to emergency medical care, which includes paramedic and nursing staff as well. The overall goal of the committee is to define the education at the level of competence in emergency medical care as a fundamental part of undergraduate medical education.

### Purpose of survey

The committee decided to collect data about the current status of undergraduate medical education in emergency medical care at German medical schools. This survey should build the foundation for further committee work, especially in finding a useful minimal standard for a nationwide curriculum in emergency medical care and in identifying research and development topics in this particular field of education.

Additionally, this survey was intended to discover weaknesses in form and content as well as applied assessment and teaching methods, and to give the participating schools feedback about their program as compared to the others.

## Methods

### Methodology and item selection

The survey was conducted in the context of the postgraduate-degree programme "Master of Medical Education-Germany" and arranged by the authors. In order to keep the questionnaire as simple as possible and yet as informative as necessary, the number of items had to be restricted to a reasonable and answerable amount. Therefore, key topics were identified; corresponding questions were formulated in these fields and divided into lists of checkboxes or open answer areas to the following topics:

▪ general information about the institution

▪ curricular structure (disciplines and workload involved, integrated teaching methods, integration of e-learning or simulation technology, used multimodal media)

▪ qualification of instructors

▪ assessment methods

▪ evaluation structure

In order to generate a comparable data structure, only compulsory course components were included in the data interpretation.

### Questionnaire

A standardized questionnaire with 70 items was sent via mail to all medical faculties in Germany (n = 38), addressed to the representative of the local emergency medical care curriculum. The questionnaire was accompanied by a cover letter from the committee that contained information about the intention of the survey as well as a reference to a website with additional information. The representatives were asked to answer the questions in the different areas by using checkboxes and open answer areas. To submit the data, each school had the choice of returning the filled out questionnaire in an enclosed self-addressed, stamped envelope, sending it via fax or returning the pdf-version of the questionnaire by e-mail (see Additional file 1).

### Data Analysis

Data are presented as mean ± SD where necessary. For analysis, statistical software SPSS 14.0 (SPSS Inc., Chicago, IL.) was used.

## Results

### General data

Overall data from 35 out of 38 of the existing medical schools in Germany were considered in the final analysis. With the exception of three (9%) of these medical schools, the local Department of Anaesthesiology is responsible for the organization of the interdisciplinary courses in emergency medical care. The course content with respect to cardiopulmonary resuscitation was predominantly (94%; n = 33) directed to the guidelines of the European Resuscitation Council (ERC) 2005 [4].

### Curriculum structure

At 49% (n = 17) of the locations, courses are organized in a longitudinal format with a minimum of two parts within the six year lasting required medical school curriculum; three faculties use a threefold concept spread over the whole curriculum. The other medical schools provide a single course lasting between one to two weeks full time. These courses were mainly scheduled in the third (31%; n = 11) or fourth year (40%; n = 14).

**Figure 1:**
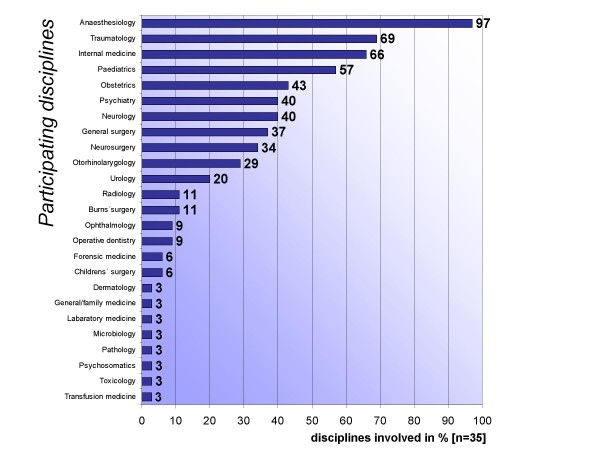
**Participating disciplines**. A listing of the participating disciplines; multiple responses were possible.

The composition of the participating disciplines varies a lot from location to location; the frequency of the participating disciplines is displayed in figure 1, whereas a minimum of two and a maximum of 14 disciplines (mean 7) are providing course components.

### Teaching methods

**Figure 2:**
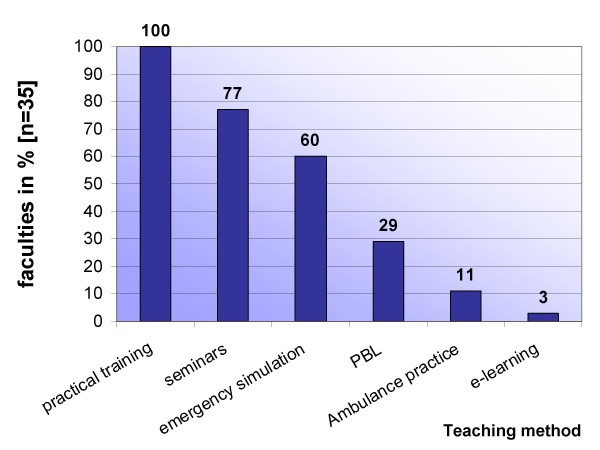
**Overview of teaching methods**. An overview of the teaching methods incorporated into medical curricula; multiple responses were possible. Refer to the text for description and definition of methods.

Lectures and practical training units are scheduled in varying composition at more than 95% (n = 34) of the locations. Regarding content, the practical sessions are focused on measures of basic life support (in the early years of study in longitudinal curricula) and advanced life support, including airway management, basics of ECG interpretation and pharmacology at nearly all medical schools (97%; n = 34). Pre-hospital or clinical trauma life support comparable to the Pre-hospital Trauma Life Support (PHTLS) or Advanced Trauma Life Support (ATLS) concepts is fixed in 26% (n = 9) of the curricula. Elements of e-learning were a mandatory part of the training in emergency medical care for only one medical school. In almost the same manner, internships in prehospital Emergency Medical Systems (EMS, ambulance service with a minimum of an 8-h-shift) were rarely mandatory (n = 4), but were available at half (46%; n = 16) of the locations as an elective opportunity. An overview to the teaching methods in general is shown in Figure 2. Problem-based learning (PBL) as teaching method is part of the curriculum at 29% (n = 10) of the medical schools, and at two of these, the curriculum is mainly PBL-based. If PBL is used as part of the curriculum, a minimum of two cases are scheduled. For the most part, the principles and the different steps of PBL follow the "Maastricht seven step approach" or the Harvard model of PBL [5]. Compulsory course components in simulation training are scheduled at 21 of the sites (60%).

### Instructor's qualification

The data on the qualifications of the faculty are very inconsistent due to fragmentary information from the institutions. If lectures are part of the curriculum, these are given by the more experienced clinicians, such as the head of the department, assistant professors or consultants; these lectures reach between 40 to 300 students per class. Instruction in smaller groups like seminars (10 to 24 participants) or practical training sessions (4 to 12 participants) is mostly managed by consultants and by experienced residents; at 46% (n = 16) of the sites, these are supported or led by student assistants (skilled as paramedics or emergency medical technicians).

The German emergency medical system provides physician-staffed ambulances nationwide with an additional qualification required; 37% (n = 13) of the medical school staff teaching in emergency medical care courses have this certification.

Five institutions (14%) explicitly specified that the members of their faculty are certified Advanced Life Support instructors of the ERC, though these five institutions provide and support ERC-ALS courses all over Germany. Two locations provide BLS and ALS with certified AHA Instructors within the curriculum.

### Assessment

**Figure 3:**
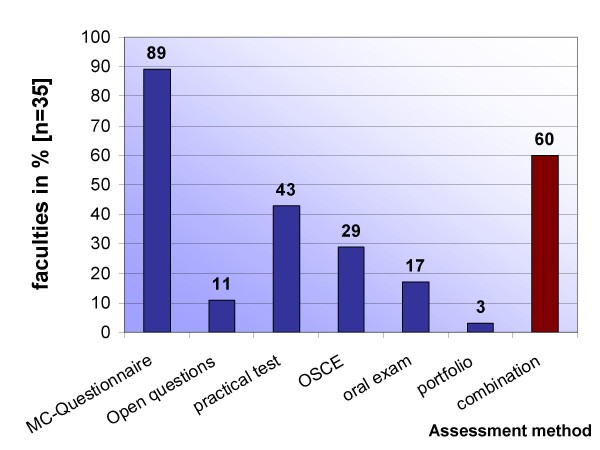
**Overview of assessment methods**. Assessment methods that are used; multiple responses were possible.

As assessment methods, multiple choice exams with a range of 15 to 70 questions are favoured (89%, n = 31), partially supplemented by open questions (31%, n = 11). Some medical schools also perform single practical tests (43%, n = 15), objective structured clinical examinations (OSCE) (29%, n = 10), oral examinations (17%, n = 6) or use portfolio (3%, n = 1). Figure 3 gives an overview of the methods used for assessment.

### Evaluation

Following evaluation of the national regulations, procedures for the scheduled courses were established at all medical schools, though with differing standards of quality. Eleven of the faculties (31%) present the results to the public via websites, 23% (n = 8) of the sites present an edited version of the results, and 23% (n = 8) do not publish their evaluation data at all.

### Optional offers

Since the new regulations were implemented, students have had to choose a certain number of compulsory subjects within the medical curriculum. A lot of different offerings are provided in the areas surrounding emergency medical care, ranging from special simulation-based training (e.g. crisis resource management) to specialized courses in disaster medicine. These topics depend on local engagement and the main focus of the organizing department.

## Discussion

Not only in the general public but also in medical professional life, there is the perception that every physician has to be able to handle critical emergency situations regardless of the location, severity of the emergency or the individual's prior experience [6]. Consequently, in 1998, the Society of American Emergency Medicine Physicians (SAEM) described three core subjects to be taught within medical school curricula: BLS skills, including the diagnosis and treatment of shock; treatment of common acute problems; and assessment of undifferentiated patients [2]. Undoubtedly, there is a need for compulsory implementation of emergency medical care content in undergraduate medical education, and in particular, many studies have revealed a low standard of necessary CPR skills in medical students and recently graduated physicians [7-9]. Jagoda et al. cited the Macy Foundation report on emergency medicine when stating "...medical school deans and faculties must ensure that every medical student has acquired the appropriate knowledge and skills to care for emergency patients" [10,11]. Accordingly, key issues in emergency medical care had to be implemented in undergraduate medical education both to fulfil the expectations of the public and society, and to invent these special core objectives as early as possible [2].

Nevertheless, few data or concepts have been described in the literature within the last ten years. In 2001, Philips and Nolan presented data from a questionnaire at UK medical schools, where compulsory BLS training was achieved for 100% of students and some sort of compulsory ALS training was implemented at most schools [6]. Finally it was unanswered, what kind of training is able to produce the necessary skills concerning ALS postulated by the Royal College of Physicians [12]. A comparable questionnaire from 2002 found that most US medical schools provide training in emergency medical care within their first two academic years, lasting from hours to weeks, and resuscitation training was scheduled in approximately 16% of the schools [13]. In addition, skills in cardiopulmonary resuscitation at the levels of BLS or ALS should be implemented in undergraduate medical education as early as possible. The 2003 publication "ILCOR Advisory Statement" summarizes this topic with a specific recommendation that "all healthcare providers should be able to demonstrate competency in the skills of BLS" and formulates that "Healthcare professionals must receive their initial training in BLS while students" [14,15].

Since new regulations were established in Germany in 2002, different local concepts for emergency medical care in undergraduate education were published, which incorporated BLS or ALS training in differing amounts [16-18]. However, no systematic and comparable nationwide survey exists to date. The present enquiry gives for the first time a nationwide overview of key elements of undergraduate education in emergency medical care and helps to update knowledge about curricula in order to describe international comparisons.

The interdisciplinary aspect of emergency medical care as a discipline is confirmed by the academic specialties involved, even though in particular cases substantial variation resulted from the local impact of special disciplines. At each site, different specialties are involved, along with a differing quantity – measured in semester periods per week – and quality with respect to the educational methods that are used. Putting these observations together, we can state that there is a sound foundation upon which the interdisciplinary development potential can be built, particularly with regard to teaching methods and their corresponding objectives. Against this background it is certainly arguable if in future emergency medicine as an own specialty of post-graduate training might be necessary or useful in order to harmonize curricular structure and content. In fact these issues are viewed very critically by existing specialties involved in emergency medical care such as anaesthesiology or traumatology.

In respect of course topics we found first of all that BLS and ALS is implemented at all faculties, but the time-frames are varying to a great extend (3 to 13 teaching units). In contrast to this diversity, as regards content the training of resuscitation in basic or advanced life support is very consistent: 94% follow the current ERC guidelines, and only two faculties are mostly AHA orientated. This is remarkable insofar as only five locations have an adequate pool of certified ERC-ALS-instructors available. Certainly this is a consequence of the fact that the German Resuscitation Council was founded as recently as 2007, and a structure for resuscitation training and qualification of instructors that is comparable to the Resuscitation Council UK or ERC does not exist to date. Undoubtedly international concepts have to be valuated and if necessary adapted and consequently implemented on a nationwide basis. However, many universities are offering high quality courses or activities with respect to methodical and didactic competencies for their own faculty, but without a widespread acceptance through all disciplines.

In spite of the uneven conditions and the lack of a sufficient number of certified instructors, most of the curricula utilize small group sessions for practical training, with four to eight participants and an instructor to student ratio of 1:3 to 1:6 depending on course content, as recommended in the 2005 ERC guidelines [19]. This recommendation already follows the ILCOR Advisory statement concerning education in resuscitation, where Chamberlain et al. proposed "moving from large-group, lecture based courses to small-group, scenario-based interactive teaching" [14,15]. Again varying concepts are used to ensure small group sessions: at some sites, students with additional qualifications such as paramedic or EMT training are trained and appointed for peer-to-peer-teaching sessions [20], whereas other locations try to make sure that only residents and consultants are integrated in practical training sessions [17].

Despite evidence about the management of severe trauma in the pre-hospital [21] or clinical setting, PHTLS-, ATLS-concepts or the program of the European Trauma Course (ETC) [22] are rarely integrated into undergraduate medical education. In one way, this is certainly caused by the lack of nationwide course concepts for professionals that compare to PHTLS or ATLS. The latter, however, is getting more relevant through the initiative of the German Society of Traumatology, which holds the rights for the courses in Germany, but should be more integrated in undergraduate education core principles. For the TEAM concept (Trauma Evaluation and Management module), Ali demonstrated that participants were better trained in providing required trauma skills [23,24].

Problem-based learning (PBL) as a teaching method was invented and used for the first time by Barrows and Tamblyn in 1976 [25]; since that time, this method found its way into newly designed as well as traditional curricula all over the world. At some medical schools, e.g. Maastricht, NL or Harvard, Boston, USA, it supports the underlying principle of the curriculum. PBL as a learning method is able to both generate knowledge to a certain extent, and to facilitate acquisition of competence with respect to problem solving strategies. Additionally, the students gained the foundation for individual life-long learning principles [5,25]. Within German medical schools, one PBL-based curriculum in emergency medical care has been published [16], but even there, elements like practical training sessions and simulations are integrated. From the educational point of view, it is certainly in question whether the problem-based learning sessions should be used as a comparable method [26]: Steadman et al. published data showing that simulation-based training was superior to PBL sessions in the acquisition of clinical skills in the field of critical care medicine [27]. A recently published editorial by Diana Wood brings this discussion to another level [28]: PBL should be accepted as one way of acquiring knowledge and problem-solving strategies as well as social skills.

Overall many contemporary and innovative teaching methods are integrated into German medical school curricula. In any case, the teaching sites have good technical features: at nearly every location, simulation technology is available to a certain extent. This is the result of a project with large-scale financial support initiated five years ago by the German Society for Anaesthesiology and Intensive Care Medicine (DGAI) to integrate simulation technology into local curricula. The main focus was to improve the quality of teaching, especially in emergency medical care, and so overall 32 Emergency Care Simulators (ECS; METI, Sarasota, FL, USA) were made available to medical schools all over the country. Besides the evidence that simulation-based training is a useful tool in medical education and is able to transfer important skills and knowledge [29], different authors have approved the use of simulation technology within undergraduate curricula [17]. Further potential operational areas like acute care in paediatric emergencies were previously presented by Eich et al. [30]; crew resource management (CRM) was presented by Müller et al. [31], resp. crisis resource management by Krüger et al. [32]. Even if every site is technologically capable of providing simulation-based training, it is important to note that necessary operational expenses such as the costs for maintenance, manpower, consumables or repairs limit the widespread implementation of this curriculum [33,34]. Additionally, no qualification standard has been set for the instructor in simulation-based training, so that we can summarize in respect of this topic: In general the didactic as well as professional qualification for teaching at the sites is very inconsistent; a standardized concept including certification compared to the generic instructor concept of the ERC is needed to enhance nationwide quality in emergency medical care in future.

A well-known model to describe medical competence is Miller's pyramid, wherein four layers of competence are defined as "knows", "knows how", "shows how" and "does" [35], and respective assessment methods are dedicated, e.g. on the level of "knows", written examinations are use with multiple-choice or open-answer questions. With respect to the assessment of CPR skills, the ILCOR-statement "education in resuscitation" postulated in 2003 "not to use written tests for CPR courses for laypersons but should be considered for healthcare professionals" [14,15]; Schuhwirth and van der Vleuten underline this statement by explaining that "one way to increase the authenticity of an assessment is to base it on a simulation of reality" [36]. On the level "shows how", Harden et al. described the so-called "OSCE"-Objective Structured Clinical Examination. Since then, OSCE has been promoted to an accepted and applied tool for the assessment of practical performance in standardized settings with prepared checklists [37,38]. An additional effect is that, as teachers know, "assessment drives learning": OSCE provokes students' learning behaviour toward practical performance and problem-solving skills as well as the individual power of judgement [39]. Despite these facts, OSCE as obligatory assessment method is utilized at one third of the schools, likely caused by the organisational and financial side effects of this "tool". Because multiple choice questions that only test the level of "knows" are used at nearly every site, the exchange of concepts and experiences necessary to bring OSCE into practice has to be facilitated. A promising concept with integration of essential emergency procedures such as BLS, ALS or diagnostic skills will be published shortly by Walcher et al.

Furthermore, the elective course offerings are often more innovative, but should be used as a preliminary stage for the widespread implementation of new concepts, and therefore communication between faculties is a necessary progression. In the future, it might be useful and interesting for applicants to see how specific locations set their priorities within the field of emergency medical care as a prime example of an interdisciplinary medical subject.

As a limitation, it is necessary to mention that we cannot rule out that in the meantime, at some locations, teaching methods such as e-learning or sessions with simulation technology or applied assessment methods have slightly changed.

However, future studies should analyse whether the different teaching and assessment methods lead to different capabilities and outcomes in the practice of emergency medical care.

## Conclusion

Emergency Medical Training in undergraduate medical education in Germany has a practical orientation, but is very inconsistently structured as well as taught. Good technical features, particularly simulation technology, are available at nearly every location, but these innovative options, as well as state-of-the-art assessment methods such as OSCE, are subject to improvement within the next curricular adjustments.

Therefore, the exchange of experiences and modern concepts among faculties and disciplines has to be promoted to simplify this process.

Based on these facts, one first step toward possible European recommendations with respect to a core curriculum in emergency medical care for undergraduate medical education has been done and should be promoted for example by the European Resuscitation Council.

Furthermore, a standardized concept including qualification as well as certification for instructors in undergraduate medical education is needed to enhance nationwide quality in emergency medical care in future.

## Abbreviations

ALS: Advanced Life Support; AHA: American Heart Association; ATLS: Advanced Trauma Life Support; BLS: Basic Life Support; CRM: crisis resource management; ERC: European Resuscitation Council; GMA: German Association for Medical Education; ILCOR: International Liaison Committee on Resuscitation; MCQ: Multiple-choice question; OSCE: objective structured clinical examination; PBL: problem-based learning; PHTLS: Pre-hospital Trauma Life Support

## Competing interests

The authors declare that they have no competing interests.

## Authors' contributions

SB, AT, MM, MA and FW had the initial idea to the study and arranged the study design and questionnaire. Literature search was performed by SB and AT, collecting of the data was performed by SB, AT, MM, MA, and FW and analysis and interpretation of the data was done by SB, AT, MM, MA, and FW. SB, AT, MM, MA and FW wrote and reviewed the manuscript before submission. All authors read and approved the final manuscript.

## Pre-publication history

The pre-publication history for this paper can be accessed here:



## Supplementary Material

Additional file 1 **Complete status-quo-questionnaire**. Displayed is the complete questionnaire. Click here for file
