# Supplementary material for: Undergraduate medical education in emergency medical care: A nationwide survey at German medical schools
Source: BMC Emerg Med. 2009 May 12;9:7. doi: 10.1186/1471-227X-9-7 (PMC2689168; doi:10.1186/1471-227X-9-7)
Supplement: Additional file 1 — Complete status-quo-questionnaire. Displayed is the complete questionnaire. [file 1471-227X-9-7-S1.pdf]

# Erhebung der Lehrsituation der notfallmedizinischen Ausbildung an Universitätskliniken in der Bundesrepublik Deutschland

- *Elektronische Version unter www.gesellschaft-medizinische-Ausbildung.de*
- *Bitte zurücksenden an sbeckers@ukaachen.de*

## Allgemein

| | | |
|---|---|---|
| **Universität** | | |
| **Ansprechpartner** | Name | |
| | e-mail | |
| | Telefon | |

Das Studium ist folgendermaßen organisiert… ❑ Semester ❑ Studienjahr ❑ Trimester

Ist die Notfallmedizin in einem eigenen "Querschnittsbereich Notfallmedizin" organisiert? ❑ Ja ❑ Nein

Wie ist die Notfallmedizin ins Curriculum eingebunden? ❑ als Block-Kurs ❑ longitudinal

❑ anders (Bitte benennen!)__________

Wer leitet/organisiert die Lehre in der Notfallmedizin?__________

Welche Fächer sind beteiligt?

❑ Allgemeinchirurgie ❑ Anästhesiologie ❑ Gynäkologie ❑ HNO
❑ Innere Medizin ❑ Neurochirurgie ❑ Neurologie ❑ Pädiatrie
❑ Psychiatrie ❑ Unfallchirugie ❑ Urologie
❑ Verbrennungschirugie ❑ Zahn-, Mund-, Kieferchirurgie

❑ Andere (Bitte benennen!)__________

Ist ein detailierter Lernzielkatalog für die Unterrichtsteile vorhanden? ❑ Ja ❑ Nein

Wenn Ja, wird er den Studierenden ausgehändigt? ❑ Ja ❑ Nein

Welcher Standard hinsichtlich der cardiopulmonalen Reanimation wird als Lehrmeinung vertreten?

❑ ERC ❑ AHA ❑ Empfehlungen BÄK ❑ keine Festlegung

❑ Andere (Bitte benennen!)__________

Gibt es eine eigene Internetpräsenz der notfallmedizinischen Lehre? ❑ Ja ❑ Nein

Wenn Ja, bitte benennen!__________

**Überblick Unterrichtsmethoden**

| | Angeboten in Semester… | Geamt-Anzahl Semesterwochen-stunden* | Gruppengröße | Dozenten je Gruppe | Anwesenheits-pflicht |
|---|---|---|---|---|---|
| Vorlesung | | | | | ❑ Ja ❑ Nein |
| Seminare | | | | | ❑ Ja ❑ Nein |
| Problem-orientiertes Lernen | | | | | ❑ Ja ❑ Nein |
| Praktikum | | | | | ❑ Ja ❑ Nein |
| Simulationstraining | | | | | ❑ Ja ❑ Nein |
| Rettungsdienst-Praktikum | | | | | ❑ Ja ❑ Nein |
| | | | | | ❑ Ja ❑ Nein |

**alternativ können auch Unterrichtsstunden (tatsächlich abgehaltene, kumulative Unterrichtsstunden à 45 min) angegeben werden; bitte ver-merken!*

## Vorlesung

| Beteiligte Fächer | ggf. Themen/Schlagworte | Semesterwochen-stunden* |
|---|---|---|
| | | |
| | | |
| | | |
| | | |

**ggf. bei Bedarf bitte Zeilen ergänzen!*
*Alternativ können auch Unterrichtsstunden (tatsächlich abgehaltene, kumulative Unterrichtsstunden à 45 min) angegeben werden; bitte ver-merken!*

## Seminar

| Beteiligte Fächer | ggf. Themen/Schlagworte | Semesterwochen-stunden* |
|---|---|---|
| | | |
| | | |
| | | |
| | | |

**ggf. bei Bedarf bitte Zeilen ergänzen!*
*Alternativ können auch Unterrichtsstunden (tatsächlich abgehaltene, kumulative Unterrichtsstunden à 45 min) angegeben werden; bitte ver-merken!*

Gibt es Seminare, die interdisziplinär gehalten werden? ❑ Ja ❑ Nein

## Problem-orientiertes Lernen (POL)

| Beteiligte Fächer | ggf. Themen/Schlagworte | Semesterwochen-stunden* |
|---|---|---|
| | | |
| | | |
| | | |
| | | |

**ggf. bei Bedarf bitte Zeilen ergänzen!*
*Alternativ können auch Unterrichtsstunden (tatsächlich abgehaltene, kumulative Unterrichtsstunden à 45 min) angegeben werden; bitte vermerken!*

Wie viele POL-Fälle werden bearbeitet?______________

Nach welchem Vorbild wird POL durchgeführt:
❑ Harvard ❑ Maastricht ❑ McMaster ❑ Andere (Bitte benennen!)________________

## Praktikum (OHNE Simulation)

| Beteiligte Fächer | ggf. Themen/Schlagworte | Semesterwochen-stunden* |
|---|---|---|
| | | |
| | | |
| | | |
| | | |

**ggf. bei Bedarf bitte Zeilen ergänzen!*
*Alternativ können auch Unterrichtsstunden (tatsächlich abgehaltene, kumulative Unterrichtsstunden à 45 min) angegeben werden; bitte vermerken!*

Gibt es Praktika, die interdisziplinär gehalten werden? ❑ Ja ❑ Nein

Wenn Ja, welche Beteiligte!________________________________________

## Simulation

| Beteiligte Fächer | ggf. Themen/Schlagworte | Semesterwochen-stunden* |
|---|---|---|
| | | |
| | | |
| | | |
| | | |

**ggf. bei Bedarf bitte Zeilen ergänzen! Alternativ können auch Unterrichtsstunden (tatsächlich abgehaltene, kumulative Unterrichtsstunden à 45 min) angegeben werden; bitte vermerken!*

Gibt es Simulation, die interdisziplinär gehalten werden? ❑ Ja ❑ Nein

Wenn Ja, welche Beteiligte!________________________________________

Welche Simulatoren werden eingesetzt?
❑ Laerdal HeartSim 200 ❑ Laerdal HeartSim 4000 ❑ Laerdal ResusciSimulator
❑ Ambu MiniMegaCodeTrainer ❑ Ambu MegaCodeTrainer
❑ Eagle ❑ Laerdal SimMan ❑ Laerdal SimBaby
❑ Meti HPS ❑ Meti ECS ❑ Meti ECS (DGAI)

❑ Andere (Bitte benennen!)________________________________________

## Ressourcen

| | Klinikchefs | Oberärzte | Fachärzte | Assistentzärzte | Pflegekräfte | Studentische Hilfskräfte |
|---|---|---|---|---|---|---|
| Vorlesung | | | | | | |
| Seminare | | | | | | |
| Problem-orientiertes Lernen | | | | | | |
| Praktikum | | | | | | |
| Simulationstraining | | | | | | |
| Rettungsdienst-Praktikum | | | | | | |
| | | | | | | |

**Bitte ankreuzen, ggf. bei Bedarf bitte Zeilen ergänzen!*

Wie wird Verbrauchsmaterial, studentische Hilfskräfte etc. finanziert?

❑ Abteilungsbudget ❑ Fakultät ❑ Studiendekanat ❑ Drittmittel

Andere (Bitte benennen!): ______________________________

Gibt es ein Anreiz-/Bonusprogramm für Lehrleistungen? ❑ Ja ❑ Nein

Wenn Ja, welche und wie hoch dotiert: ______________________________

Gibt es Preise/Auszeichnungen für Lehrprojekte? ❑ Ja ❑ Nein

Wenn Ja, welche und wie hoch dotiert: ______________________________

Sind Lehrleistungen Voraussetzung für die Habilitation? ❑ Ja ❑ Nein

Wenn Ja, welche: ______________________________

## Qualifikation der Instruktoren

Wie viele Dozenten haben welche Qualifikationen in (notfall)medizinischer Ausbildung?

| | | | | | |
|---|---|---|---|---|---|
| | ERC-BLS/AED-Provider | | AHA-BLS-Provider | | Harvard Physicians Educator |
| | ERC-ALS-Provider | | AHA-ACLS-Instructor | | Master of Medical Education (MME) |
| | ERC-ALS-Instructor | | | | eigene Teach-the-Teacher-Zertifikate |
| | ______ | | ______ | Welche: | ______ |

Ist die Tätigkeit als Notarzt Voraussetzung für den Einsatz im Unterricht? ❑ Ja ❑ Nein

## Ausbildung in der Praxis

Wenn eine praktische Ausbildung im Rettungsdienst stattfindet, auf welchem Rettungsmittel:

❑ KTW ❑ RTW ❑ NEF ❑ NAW ❑ RTH ❑ ITH

Ist ein „Logbuch" für diesen praktischen Anteil vorhanden? ❑ Ja ❑ Nein

## Prüfung

Welche Prüfungsformen kommen in der notfallmedizinischen Lehre zum Einsatz?

| | Anzahl Fragen | MCQ | Offene Fragen | MC und offene Fragen | IMPP-Fragen |
|---|---|---|---|---|---|
| **Schriftliche Prüfung** | | ❑ | ❑ | ❑ | ❑ Ja<br>❑ Nein |

| | Anzahl Fragen | Dauer | Anzahl der Prüfer | Checkliste | IMPP-Fragen |
|---|---|---|---|---|---|
| **Mündliche Prüfung** | | | | ❑ Ja<br>❑ Nein | ❑ Ja<br>❑ Nein |

| | Anzahl Stationen | Dauer | Anzahl der Prüfer | Checkliste |
|---|---|---|---|---|
| **Praktische Prüfung** | | | | ❑ Ja<br>❑ Nein |
| **OSCE** | | | | ❑ Ja<br>❑ Nein |

| **Andere *** | |
|---|---|
| **Mini-CEX** | |
| **OSLER** | |
| **Triple-Jump** | |
| | |

** Bitte ggf. Anzahl der Fragen, Prüfer, Bewertungsmaßstäbe etc. eintragen!*

## Evaluation

Wie werden die genannten Lehrveranstaltungen evaluiert:

❑ gar nicht ❑ übers Internet ❑ per SMS ❑ mit einem Fragebogen

Der Inhalt und die Form der Evaluation ist vorgegeben von . . .

❑ der eigenen Klinik ❑ der Fakultät ❑dem Studiendekanat ❑ der Fachschaft

Die Evaluation wird ausgewertet von . . .

❑ der eigenen Klinik ❑ der Fakultät ❑ dem Studiendekanat ❑ der Fachschaft

❑ extern (Bitte benennen!):______________________________

Werden die Ergebnisse der Evaluationen öffentlich gemacht (z.B. in Form einer Rangliste)?

❑ Ja ❑ Nein

Wenn Ja, wo? ❑ Per Aushang ❑ Intranet ❑ Internet

❑ Andere (Bitte benennen!):______________________
